# Novel therapeutics and emerging technology in haemostasis and thrombosis: highlights from the British society for haemostasis and thrombosis annual meeting

**DOI:** 10.3389/fcvm.2023.1225243

**Published:** 2023-09-07

**Authors:** Claire S. Whyte, Gael B. Morrow, Julia S. Gauer, Samantha J. Montague, Philip L. R. Nicolson

**Affiliations:** ^1^ Aberdeen Cardiovascular and Diabetes Centre, School of Medicine, Medical Sciences and Nutrition, Institute of Medical Sciences, University of Aberdeen, Aberdeen, United Kingdom; ^2^ Radcliffe Department of Medicine, University of Oxford, Oxford, United Kingdom; ^3^ Discovery and Translational Science Department, Leeds Institute of Cardiovascular and Metabolic Medicine, University of Leeds, Leeds, United Kingdom; ^4^ Institute of Cardiovascular Sciences, College of Medical and Dental Sciences, University of Birmingham, Birmingham, United Kingdom; ^5^ West Midlands Haemophilia Comprehensive Care Centre, University Hospitals Birmingham Foundation Trust, Birmingham, United Kingdom

**Keywords:** thrombosis, haemostasis, bleeding, coagulation, platelets, fibrinolysis

## Abstract

The 2023 annual meeting of the British Society for Haemostasis and Thrombosis (BSHT) was held in Birmingham, United Kingdom. The theme of this year's meeting was novel therapeutics and emerging technology. Here, the exciting research presented at the meeting is discussed.

## Introduction

**Figure 1:**
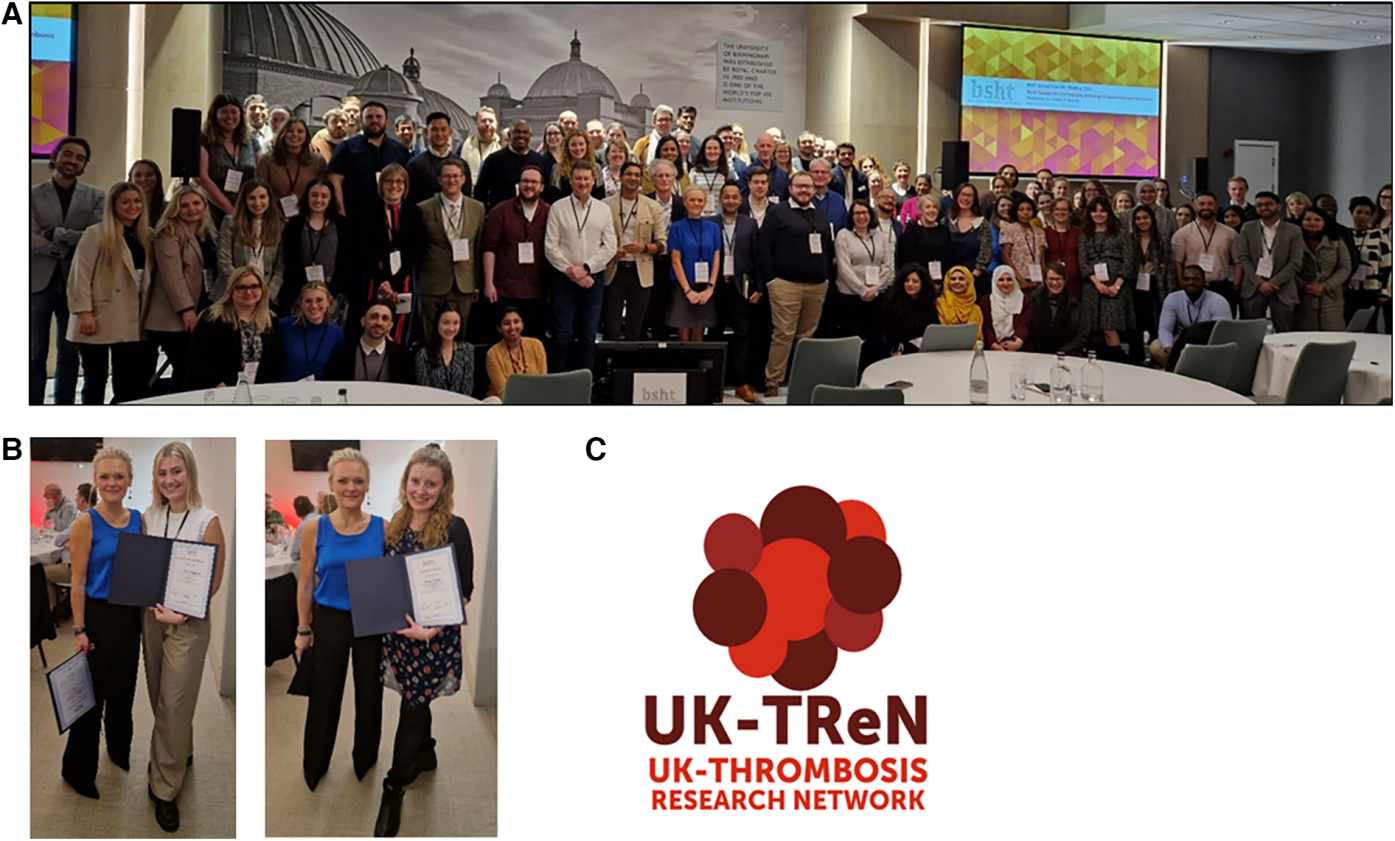
(**A**) Attendees at the British Society for Haemostasis and Thrombosis (BSHT) held its annual meeting between January 25th and 27th 2023 at the Edgbaston Park Hotel & Conference Centre, Birmingham, United Kingdom. (**B**) BSHT President Prof Nicola Mutch presenting Scientists in training winner MS Laura Mereweather (Imperial college London, UK), Best poster winner DR Joanne Clark, University of Birmingham. (**C**) UK-TReN logo designed by Steve Smith, Smith Design.

The BSHT held its annual meeting between January 25th and 27th 2023 at the Edgbaston Park Hotel & Conference Centre, Birmingham, United Kingdom. The meeting is an opportunity for academic researchers, biomedical scientists and clinicians to network and learn about recent advancements in the field of haemostasis and thrombosis. The meeting was attended by 144 delegates from 9 countries (Figure 1A) and included a selection of clinical, translational and basic science communications (Supplementary Table S1). Additional content included an Early Career Researcher (ECR) networking event and plenary speakers, industry-supported symposia, Scientist in Training, Emerging Fellows and Clinical Education sessions. Recipients of BSHT Summer Studentships also presented their projects as short oral presentations (Supplementary Table S1). There were also 14 excellent posters with a good range of topic areas, from basic science studies to novel reagent development and pre-clinical trial data.

## Early career researcher event

The meeting kicked off with an ECR networking and career development event; “Career chats on the couch.” This was an excellent opportunity for ECRs to network and listen to career development talks in an informal setting. Dr Harriet Allan (Queen Mary University of London, UK), shared her experience of successfully applying for an International Society for Thrombosis and Haemostasis (ISTH) fellowship and the unique opportunity it provides to develop your own niche and establish research independence. Professor Jules Hancox (University of Bristol, UK) gave helpful hints on preparing and interviewing for a fellowship application. Professor Anna Randi (Imperial College London, UK) shared her insights into the pros and cons of careers in academia and industry and Dr Josefin Ahnström (Imperial College London, UK) highlighted the importance of the work life balance for your mental health.

## Plenary sessions

There were six plenary speakers split over two days. Professor Ingrid Pabinger-Fasching (Medical University of Vienna, Austria) delivered a fascinating talk on “Venous thromboembolism (VTE) in cancer patients”, highlighting that the risk of thrombosis is 15% higher in cancer patients vs. non-cancer patients and the use of clinical scoring tools to predict VTE such as the Vienna CAT and Khorana scores (1). She highlighted the new CAT ESMO Guidelines (2) and significant limitations of current practice and understanding including duration of anticoagulation, how bleeding risk assessments should be incorporated and the limited awareness of oncologists about the high VTE risk in their patients. The second plenary was from Dr Roger Preston (Royal College of Surgeons in Ireland, Dublin, Ireland) who discussed “New links between immunity and blood coagulation”, with a focus on how chronic inflammatory disease predisposes individuals to increased risk of deep vein thrombosis. Dr Preston discussed the need to identify thrombotic risk and described novel techniques to analyse the procoagulant capacity of immune cells. In addition, he described mechanisms underlying how immune cells such as monocytes and macrophages may respond to inflammatory stimuli to enhance their thromboinflammatory potential.

Dr Coen Maas (University of Utrecht, The Netherlands) described the effects of Microlyse, a fusion protein targeting von Willebrand factor (vWF) (3, 4), on thrombolysis. Dr Maas discussed how the composition of the thrombus may influence thrombolysis by classic agents such as tissue plasminogen activator (tPA) and that targeting non-fibrin components could have superior effects on thrombi of different architecture. Indeed, Microlyse has been shown to be as effective as tPA in thrombi that have a high fibrin component and superior in thrombi that are platelet-rich (4).

Professor James O'Donnell (Royal College of Surgeons, Dublin, Ireland) gave an insightful presentation on bleeding disorders caused by low vWF. Professor O'Donnell discussed current pressing research questions to be addressed including understanding and management of menorrhagia, the novel functions of vWF in inflammation and the requirement for refinement of the ISTH bleeding assessment tool to account for variations due to age in females (5). Understanding the cause of reduced vWF is key, and in most patients, gene mutations do not account for low levels. Prof O'Donnell highlighted the role that reduced endothelial biosynthesis and enhanced clearance of vWF can have in contributing to disease aetiology.

Continuing on the theme of bleeding disorders, Professor Pratima Chowdary (Royal Free London, UK) presented a comprehensive overview of the advances in haemophilia treatment. This included factor replacement therapies such as Efanescoctocog alfa (6–8), rebalancing therapies such as fitusiran and marstacimab (9, 10) and factor mimetics [e.g., Mim8 (11)]. Professor Chowdary reviewed the thrombotic events reported in the tissue factor pathway inhibitor (TFPI) inhibitor trials (12, 13) and the long term outcome studies of the gene therapy trials for both haemophilia A and B (14). Professor Chowdary highlighted the changing paradigm that a zero annualised bleed rate will become the minimum standard of care and that with the huge therapeutic choice on the horizon there will be an increasing need for shared decision making and consideration for psychological support because “Normality” is a life changing event.

Dr Natalie Poulter (University of Birmingham, UK) gave an excellent overview on platelet receptor clustering and signalling. This included highlighting the use single-molecule localization microscopy (SMLM) and other advanced imaging techniques to explore platelet glycoprotein VI (GPVI) clustering and sustained signalling on immobilized collagen (15). Dr Poulter also shared recent work on the development of novel nanobody reagents against platelet receptors such as GPVI for use as potential anti-platelet therapeutics (16, 17) and new imaging tools to visualise GPVI clusters in thrombi formed under arterial shear (18).

## Scientist in training session

The Scientist in Training Session consists of presentations selected from the 5 top scoring abstracts submitted by PhD students highlighting the excellent research undertaken by ECRs within the society. This engaging session covered the role of antibodies in salmonella induced platelet aggregation, (Rachel Lamerton, University of Birmingham, UK), reprogramming of glucose metabolism in murine platelets in type 1 diabetes (Reem N Alotaibi, University of Leeds, UK), cytokine induced release of plasminogen activator inhibitor 1 in endothelial cells (Steven Humphreys, University of Aberdeen, UK) and endothelial induced changes in fibrin films that cover blood clots (Gandir Alkarithi, University of Leeds, UK). Laura Mereweather (Imperial College London, UK) was selected as the session prize winner for her impressive presentation on her development of a microfluidic model to study the initiating events of venous thrombosis (Figure 1B).

## Emerging fellows session

The Emerging Fellows Session showcases rising stars in the field who are establishing their research independence. Dr Julia Sandrin Gauer (University of Leeds, UK) discussed the potential of tyrosine kinase inhibitors and polyphenols as a novel preventative antithrombotic strategy. Dr Charis Pericleous (Imperial College London, UK) described novel models using endothelial colony forming cells which were developed to untangle the molecular mechanisms that contribute to endothelial injury in antiphospholipid syndrome. Dr Pip Nicolson (University of Birmingham, UK) described the establishment of HaemSTAR (HaemSTAR—Non-malignant Haematology Research), a UK-wide registrar-led research network funded by the National Institute of Health Research that aims to support trainee haematologists in non-malignant haematology research by supporting the development and delivery of clinical research studies. One such study was The ConNeCT study presented by Dr Rebecca Shaw (University of Liverpool, UK) who discussed the neurological complications of thrombotic thrombocytopenic purpura (TTP). Dr Shaw also highlighted her translational research using a novel assay to directly measure neutrophil extracellular traps (NETs) in plasma from patients with immune-mediated TTP. Using this assay, the presence of NETs was found to be associated with severity of neurological injury at presentation.

## Summer students session

This session showcased the work of four undergraduate students who undertook an 8–10 week studentship funded by the BSHT in summer 2022. Farieda Kassim (Biomedical Science student, Manchester Metropolitan University, UK) presented results on the haemostatic side effects of inhibiting PIM-kinase. Platelet-themed project results were presented by Caitlin Sullivan and Lily Redmond-Motteram (both Biomedical Science students from University of Birmingham, UK), who presented results on a project investigating the modulation of the recently reported S100A8/A9-induced platelet procoagulant response (19) and the use of transmission electron microscopy to analyse platelet features of patients with bleeding disorders, respectively. Finally, Lutale Metruth Kaselampao (second year medical student, University of Leeds, UK) presented data on the effect of polyphenols on fibrin clot structure and platelet activity.

## Oral communication sessions

New roles of platelets in inflammation, identification of new regulators of platelet receptors and showcasing new *in vivo* studies looking at the TFPI α anticoagulant pathway. Dr Alexander Brill (University of Birmingham, UK) kicked off this session by presenting new data on how hyperactivation of inflammasomes (NLRP3) in megakaryocyte linage induces anaemia and inflammation in mouse models. Dr Sophie Nock (Manchester Metropolitan University, UK) discussed how PIM kinases are novel regulators of platelet and megakaryocyte thromboxane A2 receptor (TPαR) and CXCR4 (20). New *in vivo* studies on Protein S and the TFPIα pathway were discussed by Dr Anastasis Petri (Imperial College London, UK) who described the role of Protein S in acting as a co-factor for human TFPIα and in controlling fibrin accumulation. Dr Parvathy Sasikumar (Imperial College London, UK) continued the story, delving into the key roles of plasma pools of human TFPIα in anticoagulant function. Dr Magdalena Gierula (Imperial College London, UK) described the role of the C-terminal tail of TFPIα on regulation of coagulation. Platelets were also the focus of the best poster prize which went to Dr Joanne Clark (University of Birmingham, UK) for her poster on their latest work on CLEC-2 clustering and CLEC-2 nanobody development (21, 22) (Figure 1B).

Novel models for the study of thrombus formation and degradation were showcased. Dr Claire Whyte (University of Aberdeen, UK) presented a novel *in vivo* murine thrombolysis model. Dr Adela Constantinescu-Bercu (University College London, UK) shared a novel microfluidic assay for monitoring TTP patients. Hosam Alden Baksamawi, (University of Birmingham, UK) showed the endothelial cell-coated microfluidic chamber he has developed which models the moving valve leaflets and hydrodynamics of a large vein (23). He convincingly demonstrated GPIbɑ-dependent development of thrombi on the valve leaflets under “back and forth” venous flow.

vWF was discussed by Daisy Jones (Imperial College London, UK) who described compounds that inhibit endothelial activation and cytokine-induced vWF release and Dr Alex Bye (St George's University of London, UK) who shared his data investigating recombinant vWF and anti-platelet therapies. Two talks were centred around thrombotic, thrombocytopenia syndromes. Dr Megan Simpson (University of Aberdeen, UK) shared her work on fibrinolysis in Vaccine-induced Immune Thrombocytopenia and Thrombosis (VITT). Dr Samantha Montague (University of Birmingham, UK) described heterogeneity of heparin-induced thrombocytopenia (HIT) and VITT donor response and how platelet-related factors may be a determining factor.

Several clinical and translational studies were presented on platelet disorders and thrombotic conditions. Catarina Isabel Loureiro Monteiro (University of Porto, Portugal) discussed the phenotypic features of a cohort of patients with inherited macrothrombocytopenia. Minka Zivkovic (University Medical Center, Utrecht, The Netherlands) gave an excellent talk on novel therapeutics reporting on the novel therapeutic bispecific antibody HMB-001 for use in Glanzmann thrombasthenia. Dr Deepa J Arachchillage (Imperial College London, UK) showed data on the hospital associated risk of thrombosis in COVID-19 (24, 25). Dr Cédric Duval (University of Leeds, UK) presented data on the fibrin coverage of thrombi taken from patients suffering from ST-elevation myocardial infraction, discussed the challenges of obtaining these samples and showed that fibrin clot coverage increased as the time from chest pain onset to thrombectomy lengthened.

## Clinical education sessions

The meeting included parallel sessions specifically for clinicians in training. Dr Karen Breen (Guy's and St Thomas’ NHS Foundation Trust, UK) discussed the investigation and treatments for some very challenging patients with antiphospholipid syndrome. The evidence (or indeed lack thereof) behind each treatment decision was discussed and this gave a very clear picture of where future research is required in this rare but potentially devastating condition. Following this, we were treated to a series of unusual case studies from clinicians and scientists-in-training. All eight presenters were competing for a case study prize which was won by Dr Marcin Lubowiecki (Oxford University Hospitals NHS Foundation Trust, UK). Dr Lubowiecki described a case of an inherited bleeding disorder that stumped all those in the audience. Whilst it was yet to be fully explained, it had a clear plan for further work up that might generate some interesting insights into the role of adrenaline on platelet GPV trafficking.

## AstraZeneca-sponsored symposium

This clinically related promotional symposium included two fantastic talks organised by AstraZenca. Professor Raza Alikhan (University Hospital Wales, UK) discussed practical application of direct oral anticoagulant reversal in the emergency setting. Dr Vinay Sehgal (University College Hospital, London, UK) described acute management of uncontrollable or life-threatening gastrointestinal bleeds including reversal of anticoagulation.

## INVENT-VTE session

Dr Lara Roberts (Kings College Hospital, UK), Dr Gill Lowe (University Hospital Birmingham, UK) and Dr Catherine Bagot (Glasgow Royal Infirmary, UK) discussed the current lack of clinical studies in VTE in the UK and how this had acted as a driver to set up UK TReN (the UK Thrombosis Research Network) (Figure 1C). The aim is to develop a network of scientists, clinicians (of multiple specialties), nurses and allied healthcare professionals to facilitate and increase national clinical and translational studies in VTE. Subsequent to the meeting, UK-TReN have joined the international INVENT-VTE network. INVENT-VTE have successfully run international collaborations that have answered important clinical questions as exemplified by the High-Low study amongst others (26–28). Anyone interested in joining UK TReN should contact uktren1@gmail.com or follow @UK_TReN.

## Concluding remarks

The inaugural BSHT annual meeting was held in the spring of 1980 at the Institute of Child Health in London, UK. Since then, the society has continued to meet annually and grown to a membership of over 300. The BSHT annual meeting 2023 delivered novel and exciting research and provided members with an excellent platform to present their research and network with new and old colleagues. This report forms part of the research topic on “Novel therapeutics and emerging technology in thrombosis and haemostasis” and highlights the vibrant research being undertaken in this area.

## Data Availability

The original contributions presented in the study are included in the article/**Supplementary Material**, further inquiries can be directed to the corresponding authors.
