# Supplementary material for: Novel therapeutics and emerging technology in haemostasis and thrombosis: highlights from the British society for haemostasis and thrombosis annual meeting
Source: Front Cardiovasc Med. 2023 Sep 7;10:1225243. doi: 10.3389/fcvm.2023.1225243 (PMC10512947; doi:10.3389/fcvm.2023.1225243)
Supplement: Supplementary file 1 [file Table1.docx]

| **Theme** | **Basic/ Translational/ Clinical** | **Presenter** | **Title** | **Session type** |
| --- | --- | --- | --- | --- |
| Bleeding disorders | Basic | Anastasis Petri (Imperial College London, UK) | Analysis of TFPIα Anticoagulant Function In Vivo | Oral Communications |
| Bleeding disorders | Basic | Parvathy Sasikumar (Imperial College London, UK) | Analysis of the Protein S - TFPIα Anticoagulant Pathway In Vivo | Oral Communications |
| Bleeding disorders | Basic | Gael Morrow (University of Oxford, UK) | FIX is localised in the vessel wall and can generate thrombin when in complex with collagen IV | Posters |
| **Bleeding disorders** | **Clinical** | **James O'Donnell (Royal College of Surgeons in Ireland, Ireland)** | **Low VWF – a bleeding disorder of unknown cause** | **Plenary** |
| **Bleeding disorders** | **Clinical** | **Pratima Chowdary (Royal Free London, UK)** | **Recent advances in haemophilia treatment** | **Plenary** |
| Bleeding disorders | Clinical | Raza Alikhan (University Hospital of Wales, UK) | Practical application of DOAC reversal in the emergency setting | AZ Sponsored Symposium |
| Bleeding disorders | Clinical | Vinay Sehgal (University College Hospital London, UK) | Acute Management of an Uncontrollable or Life-Threatening Gastrointestinal Bleeds Including Reversal of Anticoagulation | AZ Sponsored Symposium |
| Bleeding disorders | Clinical | Christina Crossette-Thambiah (Imperial College London, UK) | Acquired Haemophilia A in the COVID era – building the case for Emicizumab? | Clinical Education Session |
| Bleeding disorders | Clinical | Eman Hassan (University Hospitals of Birmingham, UK) | A challenging case of refractory ITP in and after pregnancy | Clinical Education Session |
| Bleeding disorders | Clinical | Marcin Lubowiecki (Oxford University Hospitals NHS Foundation Trust, UK) | Running low on adrenaline | Clinical Education Session |
| Bleeding disorders | Clinical | Andrew Preston (Royal Infirmary of Edinburgh, UK) | A case of coagulation testing disparity in alcohol related hepatitis | Clinical Education Session |
| Bleeding disorders | Clinical | Manmeet Singh Randhawa (Aberdeen Royal Infirmary, UK) | Therapeutic efficacy of Emicizumab in type 3 von Willebrand Disease | Clinical Education Session |
| Bleeding disorders | Clinical | Andrew Ross + Dr Rebecca Shaw (Liverpool University Hospitals NHS Foundation Trust, UK) | The FX of AL Amyloid | Clinical Education Session |
| Bleeding disorders | Clinical | Chafké Belmokhtar (Octapharma, France) | Real-world experience with a human fibrinogen concentrate: clinical data from adult and paediatric patients requiring fibrinogen for bleeding control and prevention | Posters |
| Bleeding disorders | Clinical | Christopher Reilly-Stitt (NEQAS Blood Coagulation, UK) | Post-infusion monitoring of Afstyla FVIII replacement therapy – data from the UK National External Quality assessment for Blood Coagulation (UK NEQAS BC) programme 2022 | Posters |
| Bleeding disorders | Clinical | Sophia Stanford (Basingstoke and North Hampshire Hospital, UK) | Comparison of coagulation parameters associated with fibrinogen concentrate and cryoprecipitate for treatment of bleeding in patients undergoing major cytoreductive surgery: Results from a randomised, controlled Phase 2 study | Posters |
| Bleeding disorders | Translational | Minka Zivkovic (Utrecht, The Netherlands) | HMB-001 – a novel bispecific antibody accumulating and targeting endogenous FVIIa to activated platelets supports enhanced haemostatic responses in models of Glanzmann thrombasthenia | Oral Communications |
| Bleeding disorders | Translational | Alex Bye (St George's University, UK) | Recombinant von Willebrand factor (vonvendi) mediates reversal of platelet dysfunction induced by antiplatelet agents | Oral Communications |
| Bleeding disorders | Translational | Catarina Isabel Loureiro Monteiro (University of Porto, Portugal) | Phenotypic features and genetic analysis of heterozygous and homozygous variants in glycoprotein Ib platelet alpha subunit (GP1BA), glycoprotein Ib platelet beta subunit (GP1BB) and glycoprotein IX (GP9) genes in inherited macrothrombocytopenia patients | Oral Communications |
| Bleeding disorders | Translational | Adela Constantinescu-Bercu (University College London, UK) | Acquired Glanzmann’s Thrombasthenia with IgG and IgA against activated aIIbb3 | Clinical Education Session |
| Bleeding disorders | Translational | Lily Redmond-Motteram (University of Birmingham, UK) | Analysis of platelet transmission electron microscopy in a cohort of patients with a suspected platelet-based bleeding disorder | Summer Students |
| **Platelets** | **Basic** | **Natalie Poulter (University of Birmingham, UK)** | **Platelet receptor clustering and signalling** | **Plenary** |
| Platelets | Basic | Alex Brill (University of Birmingham, UK) | NOD-, LRR- and pyrin domain-containing protein 3 (NLRP3) inflammasome hyperactivation in megakaryocyte lineage induces anaemia and enhances the inflammatory response in mice | Oral Communications |
| Platelets | Basic | Sophie Nock (Manchester Metropolitan University, UK) | Pim kinase: A novel regulator of platelet and megakaryocyte thromboxane A2 and C-X-C-R motif receptors | Oral Communications |
| Platelets | Basic | Samantha J Montague (University of Birmingham, UK) | A high degree of concordance in platelet activation between FcγRIIA stimuli | Oral Communications |
| Platelets | Basic | Rachel Lamerton (University of Birmingham, UK) | Antibodies are a key component in Salmonella induced platelet aggregation | Scientists in Training |
| Platelets | Basic | Reem N Alotaibi (University of Leeds, UK) | Reprogramming of glucose metabolism in murine platelets in type 1 diabetes | Scientists in Training |
| Platelets | Basic | Caitlin Sullivan (University of Birmingham, UK) | Investigating the expression of S100A8/A9 in platelets | Summer Students |
| Platelets | Basic | Joanne Clark (University of Birmingham, UK) | Ligand-induced clustering of CLEC-2 is governed by ligand valency, receptor density and Syk | Posters |
| Platelets | Basic | Eleyna Martin (University of Birmingham, UK) | Generation of multivalent nanobodies to probe cluster-induced platelet receptor activation | Posters |
| Platelets | Clinical | Christopher Reilly-Stitt (NEQAS Blood Coagulation, UK) | NEQAS Blood Coagulation trials platelet pools to launch a platelet EQA programme for Light Transmission Aggregometry | Posters |
| **Thrombosis** | **Basic** | **Roger Preston (Royal College of Surgeons in Ireland, Ireland)** | **New links between immunity and blood coagulation** | **Plenary** |
| Thrombosis | Basic | Julia Sandrin Gauer (University of Leeds, UK) | Pro-thrombotic clot phenotype – potential for novel therapeutic targets and repurposing of agents | Emerging Fellows |
| Thrombosis | Basic | Claire Whyte (University of Aberdeen, UK) | Localisation and function of plasminogen within the thrombus milieu | Oral Communications |
| Thrombosis | Basic | Daisy Jones (Imperial College London, UK) | Cell-based high throughput screening to identify compounds that inhibit the endothelial pro-thrombotic switch during cytokine storms | Oral Communications |
| Thrombosis | Basic | Magdalena Gierula (Imperial College London, UK) | The TFPIα C-terminal tail is essential for the synergistic enhancement of TFPIα mediated inhibition of FXa by protein S and FV-short | Oral Communications |
| Thrombosis | Basic | Hosam Alden Baksamawi (University of Birmingham, UK) | Mechanisms of platelet accumulation in an endothelium-coated elastic vein valve model of deep vein thrombosis | Oral Communications |
| Thrombosis | Basic | Laura Mereweather (Imperial College London, UK) | A microfluidic model to study the initiating events in venous thrombosis | Scientists in Training |
| Thrombosis | Basic | Ghadir Alkarithi (University of Leeds, UK) | Inflamed Endothelial Cells Reduce Clot Surface Fibrin Film Coverage | Scientists in Training |
| Thrombosis | Basic | Steven Humphreys (University of Aberdeen, UK) | Cytokines stimulate expression and release of plasminogen activator inhibitor-1 (PAI-1) from endothelial cells | Scientists in Training |
| Thrombosis | Basic | Farieda Tarek Kamaleldin Saleh Kassim (Manchester Metropolitan University, UK) | Investigating the haemostatic side effects of Pim kinase inhibitors | Summer Students |
| Thrombosis | Basic | Lutale Metruth Kaselampao (University of Leeds, UK) | The effect of polyphenols on fibrin clot structure and platelet procoagulant activity | Summer Students |
| Thrombosis | Basic | Jeries Abu-Hanna (University of Oxford, UK) | Endothelial cells delay fibrin clot lysis under static conditions in vitro | Posters |
| Thrombosis | Basic | Sophie Featherby (University of Hull, UK) | Tissue factor acts as a gauge for the level of injury by regulating the balance of expression of p16INKa and p21CIP1/WAF1 cell -cycle regulators | Posters |
| Thrombosis | Basic | Farzana Haque presented by Sophie Featherby (University of Hull, UK) | Tissue Factor (TF) and factor VII modulate the cell phenotype and are potential indicators of malignancy in pancreatic cystic lesions | Posters |
| Thrombosis | Basic | Eima Karim (Manchester Metropolitan University, UK) | Investigating endothelial cell Pim kinase as a novel anti-thrombotic target | Posters |
| **Thrombosis** | **Clinical** | **Ingrid Pabinger-Fasching (Medical University of Vienna, Austria)** | **Venous thromboembolism in cancer patients** | **Plenary** |
| Thrombosis | Clinical | Rebecca Shaw (University of Liverpool, UK) | Evolving Pathophysiological and Clinical Care Considerations in iTTP | Emerging Fellows |
| Thrombosis | Clinical | Deepa J Arachchillage (Imperial College London, UK) | Impact of thromboprophylaxis on hospital acquired thrombosis following hospital discharge in patients admitted with COVID-19 | Oral Communications |
| Thrombosis | Clinical | Gillian Lowe (University Hospital Birmingham NHS Foundation Trust, UK), Lara Roberts (King's College Hospital, UK) & Catherine Bagot (Glasgow Royal Infirmary, UK) | INVENT-VTE session | Oral Communications |
| Thrombosis | Clinical | Karen Breen (Guy's and St Thomas' NHS Foundation Trust, UK) | Common Clinical Dilemmas in Management of APS | Clinical Education Session |
| Thrombosis | Clinical | Alasdair Gray (Royal Infirmary of Edinburgh, UK) | A case report of relapse of immune-mediated thrombotic thrombocytopenic purpura after SARSCoV-2 vaccination | Clinical Education Session |
| **Thrombosis** | **Translational** | **Coen Maas (University Medical Center Utrecht, The Netherlands)** | **Microlyse - busting clots by targeting VWF** | **Plenary** |
| Thrombosis | Translational | Charis Pericleous (Imperial College London, UK) | Endothelial injury in the antiphospholipid syndrome | Emerging Fellows |
| Thrombosis | Translational | Adela Constantinescu-Bercu (University College London, UK) | A microfluidic assay to investigate thrombogenesis in TTP patients | Oral Communications |
| Thrombosis | Translational | Megan Simpson (University of Aberdeen, UK) | Fibrinolytic dysregulation in vaccine-induced immune thrombocytopenia and thrombosis (VITT) | Oral Communications |
| Thrombosis | Translational | Cedric Duval (University of Leeds, UK) | Thrombi from patients with acute myocardial infarction show fibrin film that increases with ischaemia duration | Oral Communications |
| Thrombosis | Translational | Deepa J Arachchillage (Imperial College London, UK) | Altered fibrinolytic potential in antiphospholipid syndrome | Posters |
| Thrombosis | Translational | Camille Ettelaie (University of Hull, UK) | The potential of tumour-associate tissue factor mRNA measurement as a predictor of the risk of pulmonary embolism | Posters |
| Bleeding disorders/ Platelets/Thrombosis | Clinical | Pip Nicolson (University of Birmingham, UK) | HaemSTAR: Empowering the next generation of clinical haematology researchers | Emerging Fellows |
| Bleeding disorders/ Platelets/Thrombosis | Basic | Matt J. Owen (The University of Nottingham, UK) | Mathematical models of thrombin generation: A unified approach. | Posters |

**Table 1 – Presentation themes and type.**
